# Rice proteins recognized by immunoglobulin E antibodies of patients with food allergies

**DOI:** 10.1186/2045-7022-4-S2-O6

**Published:** 2014-03-17

**Authors:** Ludmila Tuckova, Jaroslav Goliáš, Zuzana Humlová, Petr Halada, Vìra Hábová, Ivana Janatková

**Affiliations:** 1 Institute of Microbiology, The Academy of Sciences of the Czech Republic, Department of Immunology and Gnotobiology, Prague, Czech Republic; 2 1st Medical Faculty, Charles University and the General Hospital in Prague, Department of Immunology and Microbiology, Prague, Czech Republic; 3 Institute of Microbiology, The Academy of Sciences of the Czech Republic, Laboratory of Molecular Structure Characterization, Prague, Czech Republic; 4 1st Medical Faculty, Charles University and the General Teaching Hospital in Prague, Department of Immunology and Microbiology, Prague, Czech Republic

## Background

Similarity among food allergens is a great problem affecting the specificity of diagnosis and treatment of allergic patients. Rice is often considered as a hypoallergenic foodstuff, but there are several reports documenting rice-induced allergy. We have observed that 80% of patients with food (including wheat) and pollen allergies have increased IgE antibodies against rice proteins.

## Method

The extracts from wheat flour (Sulamit cultivar) or long-grain rice (Oryza sativa L.) were prepared from raw and boiled forms. The PBS-soluble and SDS-soluble proteins were analyzed by 1- or 2-DE and IgE binding components were characterized by immunoblot, basophil activation test, skin prick test and identified by MALDI-TOF mass spectrometry techniques.

## Results

We have demonstrated that boiling decrease solubility and IgE reactivity of PBS-extracted rice and wheat proteins, but in SDS-extracts this reactivity was only slightly affected. The sera of patients highly positive on the IgE immunoblot, positive in basophil activation and skin prick test with boiled rice components were used for characterizing the IgE-binding proteins separated by 1-D or 2-D electrophoresis. Employing mass spectrometry, we identified 22 rice SDS soluble proteins. Six of them were new potential rice allergens: glutelin C precursor, granule-bound starch synthase 1 protein, disulfide isomerase-like 1-1 protein, hypothetical protein OsI_13867, putative acid phosphatase precursor 1, and a protein encoded by locus Os02g0453600. These proteins retaining IgE-binding capacity also after thermal processing potentially represent important allergens in rice-containing foodstuffs. All of the identified rice proteins differed from known wheat allergens, except proteins belonging to the á amylase/trypsin-inhibitor family.

## Conclusion

We could suggest that the patients with high IgE antibodies specific to rice, positive on immunoblot, should be further tested by skin prick test with boiled rice proteins before rice recommended as a suitable diet alternative.

